# Emotion regulation strategies differentially modulate neural activity across affective prediction stages: An HD-EEG investigation

**DOI:** 10.3389/fnbeh.2022.947063

**Published:** 2022-08-05

**Authors:** Fiorella Del Popolo Cristaldi, Giovanni Mento, Giulia Buodo, Michela Sarlo

**Affiliations:** ^1^Department of General Psychology, University of Padua, Padua, Italy; ^2^Padua Neuroscience Center (PNC), University of Padua, Padua, Italy; ^3^Department of Communication Sciences, Humanities and International Studies, University of Urbino Carlo Bo, Urbino, Italy

**Keywords:** affective prediction, Emotion Regulation, ERPs, high-density EEG, S1-S2 paradigm

## Abstract

Emotion regulation (ER) strategies can influence how affective predictions are constructed by the brain (generation stage) to prearrange action (implementation stage) and update internal models according to incoming stimuli (updating stage). However, neurocomputational mechanisms by which this is achieved are unclear. We investigated through high-density EEG if different ER strategies (expressive suppression vs. cognitive reappraisal) predicted event-related potentials (ERPs) and brain source activity across affective prediction stages, as a function of contextual uncertainty. An S1-S2 paradigm with emotional faces and pictures as S1s and S2s was presented to 36 undergraduates. Contextual uncertainty was manipulated across three blocks with 100, 75, or 50% S1-S2 affective congruency. The effects of ER strategies, as assessed through the Emotion Regulation Questionnaire, on ERP and brain source activity were tested for each prediction stage through linear mixed-effects models. No ER strategy affected prediction generation. During implementation, in the 75% block, a higher tendency to suppress emotions predicted higher activity in the left supplementary motor area at 1,500–2,000 ms post-stimulus, and smaller amplitude of the Contingent Negative Variation at 2,000–2,500 ms. During updating, in the 75% block, a higher tendency to cognitively reappraise emotions predicted larger P2, Late Positive Potential, and right orbitofrontal cortex activity. These results suggest that both ER strategies interact with the levels of contextual uncertainty by differently modulating ERPs and source activity, and that different strategies are deployed in a moderately predictive context, supporting the efficient updating of affective predictive models only in the context in which model updating occurs.

## Introduction

The ability to regulate emotions is crucial to maintaining a healthy emotional life and psychological wellbeing, and to fostering successful social relationships. Let us imagine, for example, that your boss is mad and yelling at you because you didn't deliver an assignment on time. In such a situation, effectively controlling one's emotions (i.e., not yelling back at your boss) can make a difference in achieving a desirable outcome (i.e., not getting fired).

Emotion regulation (ER) is the product of the interaction between the *person* who is regulating their own emotions, the *situation* in which ER is achieved, and the regulation *strategy* they are using (Doré et al., 2016). People systematically differ in their use of ER strategies, with effects on experience and expression of emotions, interpersonal functioning, and psychological wellbeing (Gross and John, 2003). ER strategies also modulate the neurophysiological correlates of affective processing, measured in terms of both event-related potentials (ERPs) and brain activity in functional magnetic resonance imaging (fMRI; Ochsner and Gross, 2005; Hajcak et al., 2010; MacNamara et al., 2022). ER strategies can be used habitually (i.e., systematic use, stable among different situations) or circumstantially (i.e., depending on the context or on specific instructions). In the first case, they are typically measured with self-reported questionnaires such as the Emotion Regulation Questionnaire (ERQ; Gross and John, 2003). In the second case, they are studied by means of ER paradigms. In these tasks, participants are usually instructed to use specific ER strategies when presented with affective stimuli (often pictures or emotional faces; Diers et al., 2014). It is also worth noting that altered ER patterns are increasingly recognized as risk factors for the development of affective psychopathology (Cisler et al., 2010), eventually making ER crucial for promoting emotional wellbeing and physical health (Doré et al., 2016).

According to the *process model of ER* (Gross, 1998; Gross and John, 2003), ER strategies can be distinguished into antecedent-focused and response-focused. The former are mainly cognitive strategies, which develop before a full affective response is activated; whereas the latter are mainly behavioral strategies, which develop after affective responses are generated. Both types of strategies are usually automatically deployed, even though the possibility of an intentional use is not excluded (Gross and John, 2003). Two of the most common ER strategies are *cognitive reappraisal* and *expressive suppression*. Cognitive reappraisal is an antecedent-focused strategy involving the construction of an alternative representation of potentially emotion-eliciting situations in a way that changes their impact (Lazarus and Alfert, 1964; Gross and John, 2003). It has proven effective in modulating the experience and expression of emotions, eliciting greater positive emotion and lesser negative emotion, and it was found to be positively related to interpersonal functioning and wellbeing (Gross and John, 2003). Cognitive reappraisal has been found to modulate both the ERP and fMRI brain activity elicited by the reappraised affective stimuli. ERP evidence is contrasting and is primarily based on ER paradigms employing affective pictures. Some studies found that reappraisal was associated with decreased amplitudes of ERP components, such as the P2 (Pan et al., 2019), P3 (Boehme et al., 2019), and Late Positive Potential (LPP; Hajcak et al., 2010; Brudner et al., 2018; Shafir and Sheppes, 2018; Harrison and Chassy, 2019; Pan et al., 2019), especially in tasks where participants were asked to down-regulate the affective impact of the stimulus (Hajcak and Nieuwenhuis, 2006; Zhu et al., 2019). This reduced neural processing of the reappraised stimulus is consistently interpreted as supporting a less-intense emotional response. Other studies found the opposite, namely increased P2 (Wu et al., 2013) and LPP (Gan et al., 2015; Myruski et al., 2019; Cao et al., 2020) in the case of both up- and down-regulation instructions (Bernat et al., 2011; Wu et al., 2013; Baur et al., 2015; Langeslag and Surti, 2017). In this case, the authors suggest that the increased neural processing of the reappraised stimulus is due to the heightened attentional demands imposed by the reappraisal process. Other studies, some of which employ face stimuli found null effects on N170 (Herbert et al., 2013; Zhu et al., 2019; MacNamara et al., 2022), P2 (Cao et al., 2020), and LPP amplitudes (Paul et al., 2016). Interestingly, the opposite results on LPP amplitudes have been explained as depending on the level of cognitive load involved in the situation in which cognitive reappraisal has to be achieved (MacNamara et al., 2022). Reappraising a stimulus in a condition requiring more cognitive effort, e.g., when the stimulus is highly arousing, may reverse the effect of reappraisal on the LPP, eliciting larger instead of smaller amplitudes (Langeslag and Surti, 2017; MacNamara et al., 2022). fMRI evidence showed that cognitive reappraisal use activate regions, such as the bilateral middle temporal gyrus and inferior frontal gyrus (IFG), the middle frontal gyrus, and the right inferior parietal lobe (Vanderhasselt et al., 2013b). Also, it is associated with increased activity in the left medial prefrontal cortex (mPFC) and dorsolateral prefrontal cortex (dlPFC), and with decreased activity in the amygdala (Ochsner et al., 2002; Herwig et al., 2007).

Expressive suppression, instead, is a response-focused strategy involving the inhibition of the ongoing emotion-expressive behavior (Gross, 1998; Gross and John, 2003). Expressive suppression also modulates the experience and expression of emotions: it elicits lesser positive emotion and greater negative emotion, and it was found to be associated with worse interpersonal functioning and wellbeing (Gross and John, 2003). At the neurophysiological level, ERP findings are also mixed, and primarily rely on ER paradigms employing affective (especially negative) pictures. The majority of studies found that expressive suppression was associated with decreased P2 (Pan et al., 2019) and LPP amplitudes (Moser et al., 2006; Krompinger et al., 2008; Herbert et al., 2013; Li et al., 2017; Myruski et al., 2019; Pan et al., 2019; Zhu et al., 2019; MacNamara et al., 2022), indexing an attenuated attentional bias toward potentially threatening stimuli. A few other studies found increased Stimulus Preceding Negativity (SPN) prior to the stimulus to be suppressed (Shafir and Sheppes, 2018), and increased N2 (Gan et al., 2015) and LPP (Bernat et al., 2011; Paul et al., 2016) to the suppressed stimulus. These results index increased cognitive conflict when anticipating the use of expressive suppression and heightened conflict monitoring and attention allocation to the stimulus to be suppressed, respectively. Some more studies, finally, found null effects on N170 (Zhu et al., 2019) and LPP amplitudes (Brudner et al., 2018; Guex et al., 2019). fMRI evidence suggests that expressive suppression use is associated with the activation of a broad frontoparietal network composed of lateral prefrontal cortex (lPFC), mPFC, orbitofrontal cortex (OFC), supplementary motor area (SMA), and lateral-medial posterior parietal cortex (Shimamura et al., 2013; Vanderhasselt et al., 2013b), and with increased amygdala activity (Vanderhasselt et al., 2013a).

Underlying the process model of ER (Gross, 1998; Gross and John, 2003) is the conception that emotions unfold over time along three distinct stages: (1) an emotion begins with the evaluation of affective cues in the environment; (2) affective cues trigger a set of experiential, behavioral, and physiological responses; and (3), once raised, emotional responses can be modulated in various ways. This conception, although developed earlier and independently, echoes some assumptions of more recent *predictive models of emotion* (Seth and Friston, 2016; Barrett, 2017), which have re-described emotions as brain-based predictions. According to these models, people use environmental cues and their previous experience to construct affective predictions (*generation* stage). Thus, the generation stage involves the assessment of environmental cues, which is the core process of stage 1 of the process model of ER. Affective predictions are then used to anticipate incoming (and potentially relevant) stimuli, and to prepare action plans to deal with the expected situation (*implementation* stage). In stage 2 of the process model of ER, during the implementation stage, experiential, behavioral, and physiological responses are prearranged. Actual inputs are finally tested against predictions: in case of a mismatch, the discrepancy is encoded as a prediction error and used to adjust future predictions (*updating* stage). The new evidence collected during the updating stage may contribute to the redescription of affective cues, eventually modulating their impact as it happens in stage 3 of the process model of ER.

One of the most innovative contributions of predictive models of emotion is that they exclude the presence of emotion-specific brain areas, uniquely associated with each emotion category. They rather assume that affective predictions are constructed by the brain within domain-general, large-scale brain circuits supporting homeostasis and interoception (Seth and Friston, 2016; Barrett, 2017; Barrett and Satpute, 2019). Moreover, they highlight that contextual uncertainty (namely, stimuli predictability, as spontaneously inferred from the information conveyed by environmental cues) can modulate affective prediction construction (Seth and Friston, 2016; Barrett, 2017; Barrett and Satpute, 2019). This has been demonstrated by studies investigating both neural activity (Del Popolo Cristaldi et al., 2021c,d) and self-reported experience (Del Popolo Cristaldi et al., 2021a,b) during the construction of affective predictions.

Redefining emotions within a predictive framework, it becomes paramount to investigate how ER strategies can modulate the construction of affective predictions. For instance, preparing to suppress when anticipating an expected vs. unexpected affective stimulus may impact the processes developing during prediction implementation. Also, being able to effectively reappraise a stimulus (thus diminishing its impact) may serve as a more efficient model adjustment during the updating stage. However, no study to our knowledge has attempted to integrate these two perspectives by investigating whether and how the habitual use of specific ER strategies can modulate the neural correlates of affective predictions as a function of contextual uncertainty. In this way, a more comprehensive perspective on the interplay between ER and affective prediction construction may be reached, ultimately advancing knowledge in the field and providing potential clinical implications.

In light of these considerations, in the present study, we ran new analyses on a high-density EEG (HD-EEG) dataset, already collected and published (Del Popolo Cristaldi et al., 2021d). In the new analysis plan, we focused on the effects of habitual use of different ER strategies, as measured by the ERQ (Gross and John, 2003), on neural activity. In particular, we investigated whether cognitive reappraisal vs. expressive suppression predicted ERP and brain source activity across affective prediction stages, as a function of contextual uncertainty. In two previous studies, we already investigated if contextual uncertainty modulated the neural correlates of affective predictions (Del Popolo Cristaldi et al., 2021c), and whether this modulation could be mediated by individual differences in Intolerance of Uncertainty (Del Popolo Cristaldi et al., 2021d). In those studies, we employed an emotional S1-S2 paradigm (see (Mercado et al., 2008) for a review) allowing to separately investigate the three stages of affective prediction construction. S1 processing may indeed reflect the generation stage, the inter-stimulus interval (ISI) between S1 and S2 is the implementation stage, and S2 processing is the updating stage (Del Popolo Cristaldi et al., 2021c). We manipulated contextual uncertainty using three blocks with fully predictive (100%), moderately predictive (75%), or non-predictive (50%) S1-S2 affective contingency. We employed standardized emotional faces and pictures with positive (POS), negative (NEG), or neutral (NEU) valence as S1s and S2s, respectively. For each stage, in our previous studies, we focused on the following ERP components and related brain sources. As for prediction generation, we chose the N170, which reflects structural encoding and a coarse affective processing of facial expressions (Bentin et al., 1996; Blau et al., 2007). The N170 showed larger amplitudes to emotional (especially negative) facial expressions and the involvement of the right superior temporal sulcus (r-STS) as its brain source (Del Popolo Cristaldi et al., 2021c,d). For the implementation stage, we focused on the CNV developing during the ISI, which reflects the orientation of attention toward relevant stimuli and motor preparation for a potential action (Walter et al., 1964; Van Boxtel and Brunia, 1994; Chennu et al., 2013; Mento, 2013), and which is sensitive to predictive contextual factors (Chennu et al., 2013; Mento, 2013; Gómez et al., 2019). The CNV showed a larger amplitude in the non-predictive (50%) condition, and it was found to be subtended by activity within a left-lateralized network composed of anterior cingulate cortex (l-ACC), SMA (l-SMA), and dorsal posterior cingulate cortex (l-dPCC) (Del Popolo Cristaldi et al., 2021c). Lastly, as regards prediction updating, we targeted the P2, involving activity in the bilateral temporoparietal junction (l-TPJ, r-TPJ), and the LPP, involving activations within the right OFC (r-OFC) and temporal pole (r-TP). The P2 reflects early automatic attention allocation and internal models updating (Kimura and Takeda, 2015; Gómez et al., 2019), and it showed larger amplitudes to neutral pictures in all the predictive contexts (Del Popolo Cristaldi et al., 2021c,d). The LPP reflects late motivated and sustained attention allocation (Schupp et al., 2000; Olofsson et al., 2008; Hajcak et al., 2010), and it showed larger amplitudes to emotional pictures in all the predictive contexts (Del Popolo Cristaldi et al., 2021c,d). Remarkably, the above literature review on cognitive reappraisal and expressive suppression highlighted how almost all of these components are also modulated by ER strategies.

Based on our previous studies and extant ERP literature on ER, we hypothesized that (H1) no ER strategy would modulate N170 and r-STS activity during prediction generation, as N170 amplitude has been consistently shown to be insensitive to ER modulation (Herbert et al., 2013; Zhu et al., 2019; MacNamara et al., 2022). We also hypothesized that (H2) expressive suppression would predict heightened CNV amplitude (Shafir and Sheppes, 2018) and l-SMA activity (Vanderhasselt et al., 2013b) during prediction implementation, reflecting the intention to suppress (and the associated proactive inhibitory control to) the affective impact of the forthcoming S2. Lastly, considering that in our paradigm we employed highly arousing S2s, we expected that (H3) cognitive reappraisal would predict larger P2 (Wu et al., 2013) and LPP (Bernat et al., 2011; Wu et al., 2013; Baur et al., 2015; Gan et al., 2015; Langeslag and Surti, 2017; Myruski et al., 2019; Cao et al., 2020), and heightened source activations especially in the PFC (Ochsner et al., 2002; Herwig et al., 2007) during prediction updating, indexing the cognitive load of dampening the affective impact of high-arousing S2s in support of predictive models updating.

## Materials and methods

### Participants

Italian-speaking undergraduates at the University of Padua were initially screened for participation through an online survey. The survey evaluated the inclusion criteria for the study: absence of neurological and/or psychiatric disorders; normal or corrected-to-normal vision; no medication taken; right-handedness, as assessed by the Edinburgh Handedness Inventory (Oldfield, 1971); and no high blood-injection-injury fear, as assessed by the Fear Survey Schedule (Wolpe and Lang, 1964). Since some experimental stimuli depicted gory scenes, the last criterion was included in respect of ethical reasons to discard highly fearful participants (i.e., participants who scored 4 -on a 0-4 score- on items concerning blood, injuries).

Thirty-six right-handed participants (16 males, age: *M* = 23.25, *SD* = 1.85, range = 20–29) met the inclusion criteria. All participants signed an informed consent and took part in the study as volunteers. The sample size was based on previous ERP research studying ER within S1-S2 paradigms (Brudner et al., 2018). A *post-hoc* power analysis (α = 0.05, η^2^ =0.02) showed that the final sample size (*N* =36) allowed to reach an average power of 0.541. All experimental procedures were approved by the Ethical Committee for the Psychological Research of the University of Padua (protocol no. 2859) and were conducted in accordance with the Declaration of Helsinki.

### Stimulus material

Individual differences in the habitual use of ER strategies were assessed with the ERQ (Gross and John, 2003), administered in its Italian validated adaptation (Balzarotti et al., 2010). The ERQ is a 10-item self-report questionnaire. It consists of two subscales, corresponding to cognitive reappraisal (six items) and expressive suppression (four items) strategies. Each item is rated on a 7-point-Likert scale (from “strongly disagree” to “strongly agree”), and the mean ratings for each strategy constitute the reappraisal and suppression scores (range 1–7 each). Means (*M*) and standard deviations (*SD*) of the cognitive reappraisal and expressive suppression scores in our sample were *M* = 4.97, *SD* = 1.37 and *M* = 3.52, *SD* = 1.41, respectively.

The task consisted of a computerized S1-S2 passive viewing paradigm (already described in Del Popolo Cristaldi et al., 2021c). In experimental trials, 24 different colored faces (four male and four female Caucasian models, each posing fearful, happy and neutral expressions) from the NimStim Set of Facial Expressions (Tottenham et al., 2009) were employed as S1s. S2s were 120 colored pictures (40 high-arousing negative, 40 high-arousing positive, and 40 low-arousing neutral) from the International Affective Picture System (IAPS; Lang et al., 2008). In four practice trials, two additional faces (1 male and 1 female, posing angry and surprised expressions) and four additional pictures (2 low-arousing positive and 2 low-arousing negative) were employed as S1s and S2s, respectively. NimStim and IAPS picture numbers, sorted by valence, are listed in Supplementary Table 1. Positive and negative pictures did not differ for mean arousal standardized ratings [*M* = 6.43 and 6.44, *SD* = 0.46 and 0.62, respectively; *t*_(93)_ = 0.12, *p* = 0.9].

### Procedure

Before arrival in the laboratory, all participants answered an online version of the ERQ (Gross and John, 2003; Balzarotti et al., 2010). Upon arrival, participants were seated in a dimly lit room, at a 90 cm viewing distance from a computer screen (24-inch, 1,280 × 1,024 px resolution). They received information about EEG montage and the experimental task. Then, an elastic 128-channel HD-EEG net was applied.

**Figure 1:**
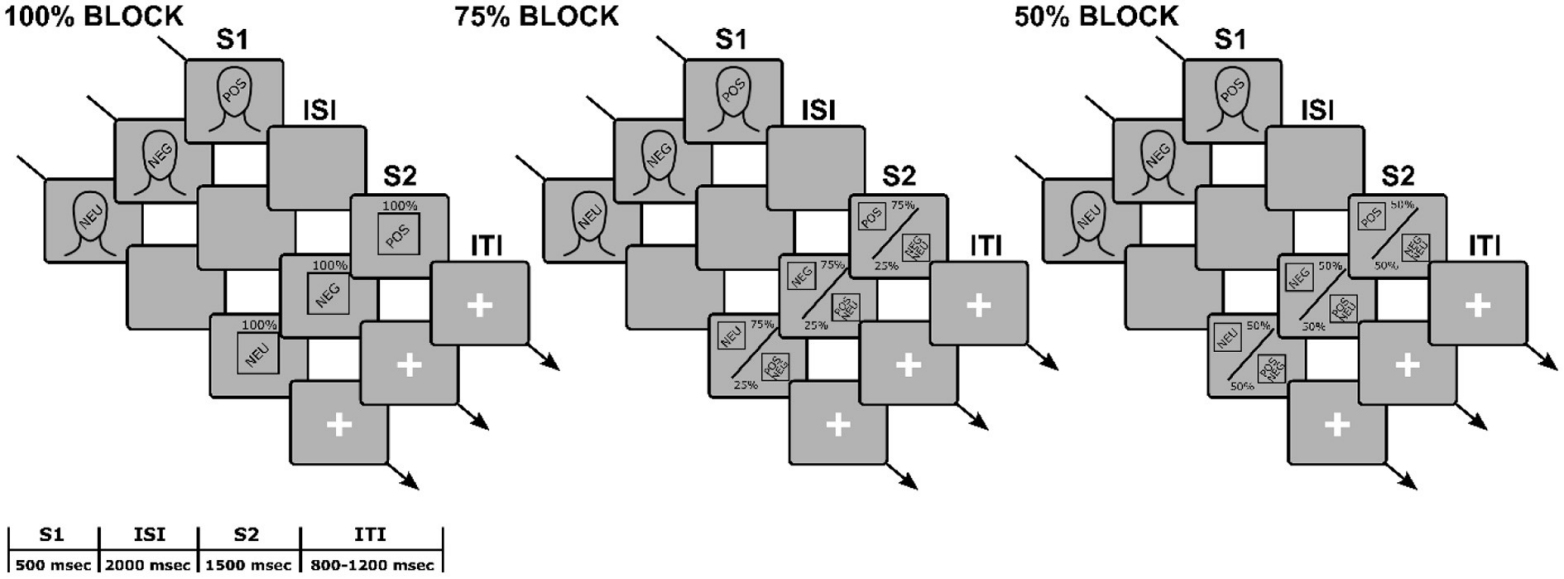
Schematic representation of the experimental paradigm.

After a 5-min adaptation period, the S1-S2 paradigm started (see Figure 1 for a schematic representation). S1s and S2s were presented through E-prime software (Schneider et al., 2010). All stimuli were presented in their original size, in the center of the screen against a black background. The presentation order was pseudo-random, with a maximum of three subsequent S1-S2 same-valence pairings as a constraint. At the beginning of the S1-S2 task, participants read the instructions presented on the screen at their own pace. They were told that they would see a face followed by a picture and that they only had to look at the screen, trying to move as little as possible. A practice session followed, including two congruent (i.e., same-valence S1-S2 pairs) and two incongruent (i.e., different-valence S1-S2 pairs) trials. The task included a total of 360 trials. Each trial began with an S1, presented for 500 ms. A fixed ISI of 2,000 ms followed, in which the screen remained black. Then an S2 was presented for 1,500 ms. The inter-trial interval (ITI) randomly varied between 800 and 1,200 ms. During the ITI, a white fixation cross against a black background was displayed.

We manipulated S1-S2 affective congruency through three blocks of 120 trials each. In the 100% block, S1 was fully predictive of S2 valence (i.e., S1 and S2 were always congruent). In the 75% block, S1 was moderately predictive of S2 valence (i.e., S1 and S2 were congruent in 75% of the trials). In the 50% block, S1 was unpredictive of S2 valence (i.e., S1 was randomly followed by a positive, negative, or neutral S2). Blocks were presented seamlessly with self-paced brief random breaks and a between-subjects counterbalanced order. Participants were left uninstructed about the different between-blocks S1-S2 probabilistic ratio.

At the end of the task, the experimenter orally asked each participant if they had caught any relationship between the face and the picture during the S1-S2 paradigm. This interview aimed to ensure that all participants had remained unaware of the exact S1-S2 probabilistic ratios of the three blocks. No participant reported to have caught the exact blocks' ratios. At the end of the experimental session, each participant was informed about the research objectives and thanked for their participation.

Example sequence of events and their duration for a trial, according to the *block* (100, 75, 50%), *S1 valence* (POS, NEG, NEU), and *S2 valence* (POS, NEG NEU). In the 100% block, the face (S1) was followed by a picture (S2) of the same valence in 100% of the trials; in the 75% block, the S1 was followed by an S2 of the same valence in 75% of the trials, and of different valence in 25% of the trials; in the 50% block, the S1 was followed by an S2 of the same valence in 50% of the trials, and of different valence in the other 50% of the trials. Participants were asked to passively view the stimuli while their EEG signal was recorded. The text is not drawn to scale.

### EEG recordings and pre-processing

The EEG data analyzed in this study were derived from our previous study (Del Popolo Cristaldi et al., 2021d) and they are publicly available (https://doi.org//10.6084/m9.figshare.13560569). During the task, EEG was continuously recorded with a Geodesic HD-EEG System (EGI^®^ GES-300), through a pre-cabled 128-channel HydroCel Geodesic Sensor Net (HCGSN-128). All electrodes were referenced online to the vertex. Scalp voltages were amplified through a 24-bit DC amplifier. The sampling rate was 500 Hz. The impedance was kept below 60 kΩ for each sensor.

EEG recordings were preprocessed using the MATLAB toolbox EEGLAB 14.1.2b (Delorme and Makeig, 2004). Pre-processing steps, as described by Del Popolo Cristaldi et al. (2021c), included downsampling at 250 Hz, and filtering with a digital band-pass filter (0.01–40 Hz,−6dB - Hamming windowed sinc finite impulse response filter, filter order = 82,500). Then, continuous EEG was segmented into 4,500 ms epochs, from 500 ms before to 4,000 ms after S1 onset. The signal was baseline corrected to two different baselines: from 500 ms before to the onset of S1 for S1-ERPs; from 200 ms before to the onset of S2 for S2-ERPs. Epochs were digitally inspected through the TBT EEGLAB plug-in and applied to electrodes from E40 to E100 (frontal electrode sites were excluded because no eye blinks/movements correction was yet applied). The TBT algorithm performed an automatic rejection of epochs and interpolation of channels on an epoch-by-epoch basis: channels that exceeded a differential average amplitude of ±150 μV on more than 30% of all epochs were marked as bad, excluded, and subsequently interpolated with the spherical spline interpolation method (Perrin et al., 1989; Ferree, 2006). Epochs having more than 10 bad channels were also excluded. Artifact-reduced data were then subjected to Independent Component Analysis (ICA; Stone, 2002) using the Infomax algorithm (Bell and Sejnowski, 1995). All independent components were visually inspected to discard those related to eye blinks, eye movements, heartbeat, and muscular signals, according to their morphology and scalp distribution. The remaining components were projected back to the electrode space. Epochs were further visually inspected and residual artifact-contaminated trials were rejected. Experimental conditions did not differ for the final number of epochs (see Supplementary Table 2). Data were finally re-referenced to the average of all electrodes. Individual average and grand average ERPs were computed for all experimental conditions, applying a weighted average in order to control for any potential unbalanced number of epochs per condition (Kotowski et al., 2019).

### ERPs and brain source activity

ERPs and brain source activity used as predicted measures in this study were derived from our previous research (Del Popolo Cristaldi et al., 2021c,d). For each stage of affective prediction construction, ERPs were computed as the mean voltage amplitude in the following time windows and electrode clusters. For prediction *generation*, we measured the N170 (140–180 ms to S1 onset) from an occipital cluster (E70, E74, E75, E81, E82, E83). For prediction *implementation*, we targeted both early (1,500–2,000 ms to S1 onset) and late CNV (2,000–2,500 ms to S1 onset), as measured from a left-central cluster (E40, E41, E42, E46, E47). For prediction *updating*, we targeted the P2 (200–300 ms to S2 onset), as measured from a parietal cluster (E67, E71, E72, E75, E76, E77), and both early (400–600 ms to S2 onset) and late LPP (600–800 ms to S2 onset), as measured from a parietal cluster (E60, E61, E62, E67, E72, E77, E78, E85).

For each ERP component, source map vertices were clustered in the following regions of interest (ROI), according to our previous research (Del Popolo Cristaldi et al., 2021c,d): r-STS as the estimated brain source of the N170; l-ACC, l-SMA, and l-dPCC as the sources of the CNV; l-TPJ and r-TPJ as the sources of the P2; and r-OFC and r-TP as the sources of the LPP. Absolute values of each ROI were time-averaged from the pertaining ERP time window, extracted for each participant, and transformed using a natural logarithm.

### Data analysis

The study has a 3 (*block*: 100, 75, 50%) × 3 (S1 or S2 *valence*: POS, NEG, NEU) within-subjects design.

To investigate whether ERQ reappraisal and suppression scores predicted the ERP and brain source activity during the S1-S2 task, we fitted the following linear mixed-effects models (LMMs; R package: lme4; Bates et al., 2015). For each DV (averaged ERPs and ROIs activity), we fitted separate LMMs with *block, valence, ERQ reappraisal* scores (mean centered), *ERQ suppression* scores (mean centered), and their interactions as fixed factors and individual random intercept.

Model effects were evaluated using *F*-test and *p*-values, calculated *via* Satterthwaite's degrees of freedom method (α = 0.05, R package: lmerTest; Kuznetsova et al., 2017). For each model, we report marginal and conditional *R*^2^ as estimated by Nakagawa et al. (2017). The slopes of the ERQ trends for each level of the factors (block, valence) were estimated. *Post-hoc* pairwise comparisons between the slopes of the ERQ scores trend for each level of the fixed factors were tested using estimated marginal means (EMMs) contrasts, Tukey-adjusted for multiple comparisons (R package: emmeans; Lenth, 2020). For the sake of clarity, and to avoid redundant results from our previous findings (Del Popolo Cristaldi et al., 2021c,d), only effects involving ERQ scores, slopes statistically different from 0, and significant *post-hoc* contrasts are reported and commented in the Results, while all remaining effects are reported in the Supplementary material.

## Results

### Prediction generation stage

Models on N170 and r-STS activity are summarized in Table 1. As hypothesized (H1), no significant main or interaction effects of the ERQ reappraisal and suppression subscales were observed.

**Table 1:** ANOVA table of models in the prediction generation stage.

**DV**	**Effect**	**SS**	**df_num_**	**df_den_**	***F***	***p***	**Partial η^2^**
N170	Block	1.75	2	256	0.91	0.402	0.007
	Valence	23.19	2	256	12.12	<0.001	0.086
	ERQ reappraisal	1.99	1	32	2.08	0.158	0.061
	ERQ suppression	0.47	1	32	0.49	0.489	0.015
	Block × valence	5.37	4	256	1.40	0.233	0.021
	Block × ERQ reappraisal	1.87	2	256	0.98	0.378	0.008
	Valence × ERQ reappraisal	1.41	2	256	0.74	0.479	0.006
	Block × ERQ suppression	2.09	2	256	1.09	0.338	0.008
	Valence × ERQ suppression	0.55	2	256	0.29	0.750	0.002
	ERQ reappraisal × ERQ suppression	0.00	1	32	0.00	0.950	0.000
	Block × Valence × ERQ reappraisal	2.11	4	256	0.55	0.698	0.009
	Block × Valence × ERQ suppression	1.85	4	256	0.48	0.748	0.008
	Block × ERQ reappraisal × ERQ suppression	1.23	2	256	0.64	0.528	0.005
	Valence × ERQ reappraisal × ERQ suppression	8.63	2	256	2.85	0.059	0.006
	Block × valence × ERQ reappraisal × ERQ suppression	6.00	4	256	0.99	0.412	0.024
	*R*^2^ marginal/conditional	0.08/0.88
r-STS	Block	0.98	2	256	0.69	0.503	0.005
	Valence	1.75	2	256	1.23	0.295	0.009
	ERQ reappraisal	0.10	1	32	0.14	0.714	0.004
	ERQ suppression	0.07	1	32	0.10	0.752	0.003
	Block × valence	0.56	4	256	0.20	0.940	0.003
	Block × ERQ reappraisal	0.74	2	256	0.52	0.593	0.004
	Valence × ERQ reappraisal	0.26	2	256	0.19	0.830	0.001
	Block × ERQ suppression	1.84	2	256	1.29	0.276	0.010
	Valence × ERQ suppression	3.31	2	256	2.33	0.100	0.018
	ERQ reappraisal × ERQ suppression	1.12	1	32	1.58	0.218	0.047
	Block × valence × ERQ reappraisal	1.65	4	256	0.58	0.677	0.009
	Block × valence × ERQ suppression	2.10	4	256	0.74	0.567	0.011
	Block × ERQ reappraisal × ERQ suppression	0.32	2	256	0.23	0.799	0.002
	Valence × ERQ reappraisal × ERQ suppression	3.35	2	256	2.35	0.097	0.018
	Block × valence × ERQ reappraisal × ERQ suppression	2.09	4	256	0.73	0.569	0.011
	*R*^2^ marginal/conditional	0.06/0.69

For each dependent variable (DV) and effect of interest, we report the sum of squares (SS), numerator (df_num_), and denominator (df_den_) degrees of freedom, F-test and relative p-values, and partial η^2^.

### Prediction implementation stage

**Figure 2:**
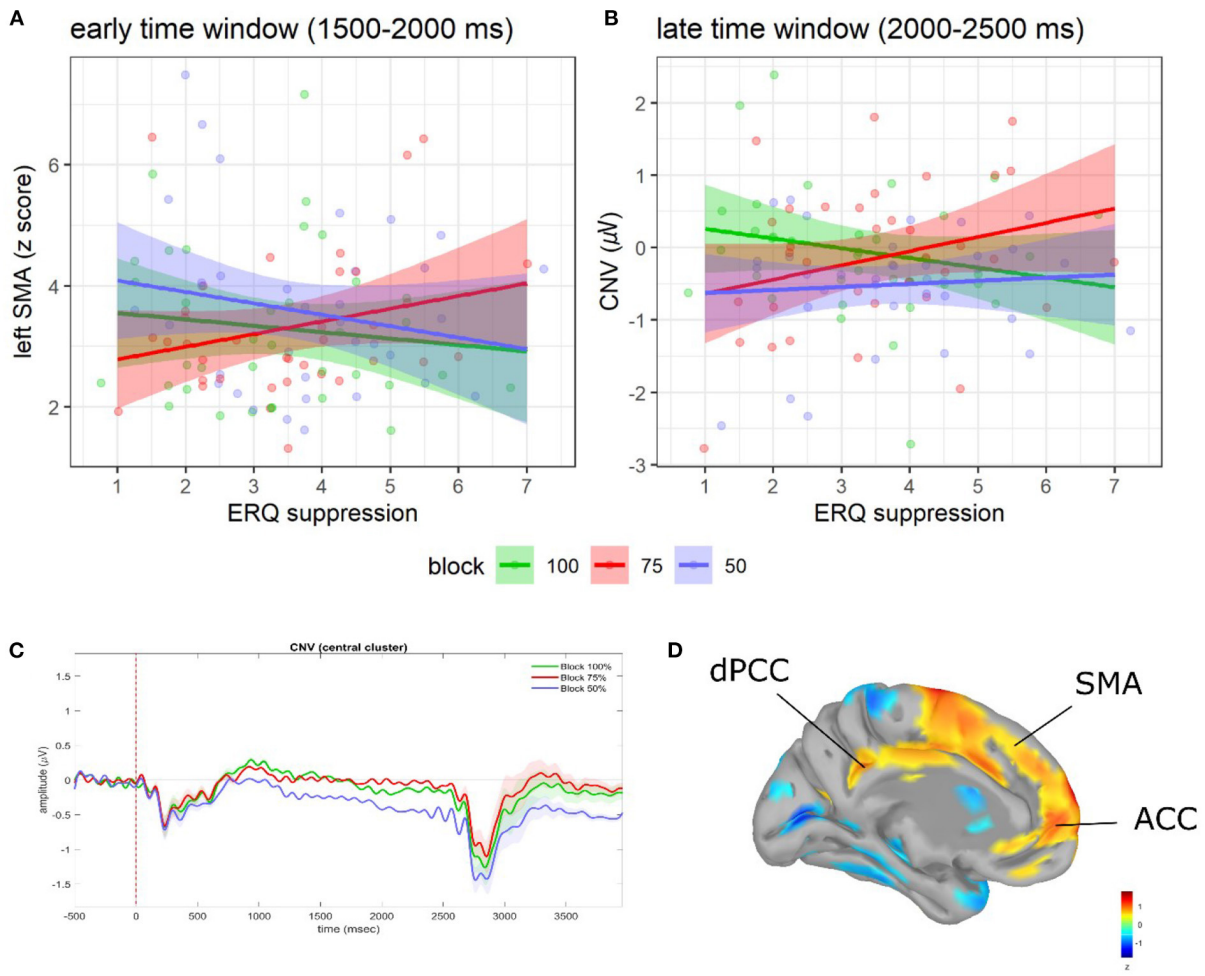
Regression plots of ERQ suppression scores on **(A)** early l-SMA activity and **(B)** late CNV amplitude as a function of blocks. Shaded areas denote the 95% CI. Points represent individual observations. Grand average ERP waveforms during ISI as a function of blocks **(C)**. Shaded areas denote SE. For visualization purposes, waveforms were low-pass re-filtered at 10 Hz. Adapted from Del Popolo Cristaldi et al. (2021d). Brain activity (*z*-score) of the l-ACC, l-SMA, and l-dPCC **(D)**.

Models on CNV, l-ACC, l-SMA, and l-dPCC activity are summarized in Table 2, Figure 2, and Supplementary Table 3.

**Table 2:** ANOVA of models in the prediction implementation stage.

**DV**	**Effect**	**SS**	**df_num_**	**df_den_**	***F***	***p***	**Partial η^2^**
Early CNV	Block	6.39	2	256	2.52	0.082	0.019
	Valence	3.21	2	256	1.27	0.284	0.010
	ERQ reappraisal	2.24	1	32	1.77	0.193	0.052
	ERQ suppression	0.02	1	32	0.02	0.900	0.000
	Block × valence	1.97	4	256	0.39	0.817	0.006
	Block × ERQ reappraisal	3.43	2	256	1.35	0.260	0.010
	Valence × ERQ reappraisal	3.83	2	256	1.51	0.223	0.012
	Block × ERQ suppression	2.39	2	256	0.94	0.391	0.007
	Valence × ERQ suppression	4.73	2	256	1.87	0.157	0.014
	ERQ reappraisal × ERQ suppression	1.95	1	32	1.54	0.223	0.046
	Block × valence × ERQ reappraisal	1.34	4	256	0.26	0.900	0.004
	Block × valence × ERQ suppression	8.61	4	256	1.70	0.151	0.026
	Block × ERQ reappraisal × ERQ suppression	1.19	2	256	0.47	0.626	0.004
	Valence × ERQ reappraisal × ERQ suppression	3.67	2	256	1.45	0.237	0.011
	Block × valence × ERQ reappraisal × ERQ suppression	3.87	4	256	0.76	0.550	0.012
	*R*^2^ marginal/conditional	0.11/0.20					
Early l-ACC	Block	13.31	2	256	1.96	0.142	0.015
	Valence	2.84	2	256	0.42	0.659	0.003
	ERQ reappraisal	3.83	1	32	1.13	0.295	0.034
	ERQ suppression	0.04	1	32	0.01	0.912	0.000
	Block × valence	14.63	4	256	1.08	0.367	0.017
	Block × ERQ reappraisal	0.72	2	256	0.11	0.900	0.001
	Valence × ERQ reappraisal	1.15	2	256	0.17	0.844	0.001
	Block × ERQ suppression	2.85	2	256	0.42	0.657	0.003
	Valence × ERQ suppression	0.14	2	256	0.02	0.980	0.000
	ERQ reappraisal × ERQ suppression	1.78	1	32	0.52	0.474	0.016
	Block × valence × ERQ reappraisal	4.59	4	256	0.34	0.852	0.005
	Block × valence × ERQ suppression	11.64	4	256	0.86	0.489	0.013
	Block × ERQ reappraisal × ERQ suppression	16.06	2	256	2.37	0.096	0.018
	Valence × ERQ reappraisal × ERQ suppression	2.83	2	256	0.42	0.659	0.003
	Block × valence × ERQ reappraisal × ERQ suppression	17.13	4	256	1.26	0.285	0.019
	*R*^2^ marginal/conditional	0.07/0.32					
Early l-SMA	Block	6.72	2	256	1.25	0.288	0.010
	Valence	2.38	2	256	0.44	0.642	0.003
	ERQ reappraisal	6.98	1	32	2.60	0.117	0.075
	ERQ suppression	0.13	1	32	0.05	0.825	0.002
	Block × valence	10.36	4	256	0.96	0.428	0.015
	Block × ERQ reappraisal	5.92	2	256	1.10	0.334	0.009
	Valence × ERQ reappraisal	2.92	2	256	0.54	0.582	0.004
	Block × ERQ suppression	17.50	2	256	3.26	0.040	0.025
	Valence × ERQ suppression	4.96	2	256	0.92	0.399	0.007
	ERQ reappraisal × ERQ suppression	0.01	1	32	0.00	0.959	0.000
	Block × valence × ERQ reappraisal	0.80	4	256	0.07	0.990	0.001
	Block × valence × ERQ suppression	11.00	4	256	1.02	0.396	0.016
	Block × ERQ reappraisal × ERQ suppression	10.57	2	256	1.97	0.142	0.015
	Valence × ERQ reappraisal × ERQ suppression	5.15	2	256	0.96	0.385	0.007
	Block × valence × ERQ reappraisal × ERQ suppression	6.44	4	256	0.60	0.664	0.009
	*R*^2^ marginal/conditional	0.09/0.25					
Early l-dPCC	Block	0.98	2	256	0.24	0.789	0.002
	Valence	1.06	2	256	0.26	0.775	0.002
	ERQ reappraisal	2.36	1	32	1.14	0.294	0.034
	ERQ suppression	0.83	1	32	0.40	0.532	0.012
	Block × valence	1.63	4	256	0.20	0.940	0.003
	Block × ERQ reappraisal	7.06	2	256	1.71	0.183	0.013
	Valence × ERQ reappraisal	6.70	2	256	1.62	0.200	0.012
	Block × ERQ suppression	6.68	2	256	1.61	0.201	0.012
	Valence × ERQ suppression	0.98	2	256	0.24	0.789	0.002
	ERQ reappraisal × ERQ suppression	6.29	1	32	3.04	0.091	0.087
	Block × valence × ERQ reappraisal	4.76	4	256	0.58	0.680	0.009
	Block × valence × ERQ suppression	3.99	4	256	0.48	0.748	0.007
	Block × ERQ reappraisal × ERQ suppression	15.08	2	256	3.65	0.027	0.028
	Valence × ERQ reappraisal × ERQ suppression	1.71	2	256	0.41	0.661	0.003
	Block × valence × ERQ reappraisal × ERQ suppression	2.87	4	256	0.35	0.846	0.005
	*R*^2^ marginal/conditional	0.09/0.31					
Late CNV	Block	12.27	2	256	4.06	0.018	0.031
	Valence	6.03	2	256	1.99	0.138	0.015
	ERQ reappraisal	2.38	1	32	1.58	0.218	0.047
	ERQ suppression	0.28	1	32	0.19	0.668	0.006
	Block × valence	5.57	4	256	0.92	0.452	0.014
	Block × ERQ reappraisal	3.43	2	256	1.13	0.324	0.009
	Valence × ERQ reappraisal	4.69	2	256	1.55	0.213	0.012
	Block × ERQ suppression	11.10	2	256	3.67	0.027	0.028
	Valence × ERQ suppression	3.26	2	256	1.08	0.342	0.008
	ERQ reappraisal × ERQ suppression	2.16	1	32	1.43	0.240	0.043
	Block × valence × ERQ reappraisal	3.66	4	256	0.61	0.659	0.009
	Block × valence × ERQ suppression	14.06	4	256	2.33	0.057	0.035
	Block × ERQ reappraisal × ERQ suppression	2.49	2	256	0.83	0.439	0.006
	Valence × ERQ reappraisal × ERQ suppression	8.63	2	256	2.85	0.059	0.022
	Block × valence × ERQ reappraisal × ERQ suppression	6.00	4	256	0.99	0.412	0.015
	*R*^2^ marginal/conditional	0.14/0.26					
Late l-ACC	Block	30.29	2	256	3.23	0.041	0.025
	Valence	1.74	2	256	0.19	0.831	0.001
	ERQ reappraisal	6.05	1	32	1.29	0.264	0.039
	ERQ suppression	0.18	1	32	0.04	0.847	0.001
	Block × valence	15.62	4	256	0.83	0.505	0.013
	Block × ERQ reappraisal	12.49	2	256	1.33	0.265	0.010
	Valence × ERQ reappraisal	2.95	2	256	0.32	0.730	0.002
	Block × ERQ suppression	0.73	2	256	0.08	0.925	0.001
	Valence × ERQ suppression	0.41	2	256	0.04	0.957	0.000
	ERQ reappraisal × ERQ suppression	1.64	1	32	0.35	0.558	0.011
	Block × valence × ERQ reappraisal	8.39	4	256	0.45	0.774	0.007
	Block × valence × ERQ suppression	21.89	4	256	1.17	0.325	0.018
	Block × ERQ reappraisal × ERQ suppression	11.14	2	256	1.19	0.306	0.009
	Valence × ERQ reappraisal × ERQ suppression	6.37	2	256	0.68	0.508	0.005
	Block × valence × ERQ reappraisal × ERQ suppression	15.41	4	256	0.82	0.512	0.013
	*R*^2^ marginal/conditional	0.07/0.34					
	*R*^2^ marginal/conditional	0.09/0.25					
Late l-SMA	Block	7.13	2	256	1.01	0.364	0.008
	Valence	4.51	2	256	0.64	0.527	0.005
	ERQ reappraisal	11.66	1	32	3.32	0.078	0.094
	ERQ suppression	0.47	1	32	0.14	0.716	0.004
	Block × valence	10.62	4	256	0.76	0.555	0.012
	Block × ERQ reappraisal	10.22	2	256	1.45	0.235	0.011
	Valence × ERQ reappraisal	3.93	2	256	0.56	0.572	0.004
	Block × ERQ suppression	13.33	2	256	1.90	0.152	0.015
	Valence × ERQ suppression	3.56	2	256	0.51	0.603	0.004
	ERQ reappraisal × ERQ suppression	0.88	1	32	0.25	0.620	0.008
	Block × valence × ERQ reappraisal	3.75	4	256	0.27	0.899	0.004
	Block × valence × ERQ suppression	16.56	4	256	1.18	0.320	0.018
	Block × ERQ reappraisal × ERQ suppression	13.45	2	256	1.92	0.149	0.015
	Valence × ERQ reappraisal × ERQ suppression	5.90	2	256	0.84	0.433	0.007
	Block × valence × ERQ reappraisal × ERQ suppression	7.09	4	256	0.50	0.732	0.008
	*R*^2^ marginal/conditional	0.09/0.28					
Late l-dPCC	Block	5.07	2	256	0.95	0.388	0.007
	Valence	1.27	2	256	0.24	0.788	0.002
	ERQ reappraisal	3.97	1	32	1.49	0.231	0.044
	ERQ suppression	0.90	1	32	0.34	0.565	0.010
	Block × valence	2.67	4	256	0.25	0.910	0.004
	Block × ERQ reappraisal	14.40	2	256	2.70	0.069	0.021
	Valence × ERQ reappraisal	6.28	2	256	1.18	0.310	0.009
	Block × ERQ suppression	2.64	2	256	0.49	0.611	0.004
	Valence × ERQ suppression	1.67	2	256	0.31	0.732	0.002
	ERQ reappraisal × ERQ suppression	8.25	1	32	3.09	0.088	0.088
	Block × valence × ERQ reappraisal	2.73	4	256	0.26	0.906	0.004
	Block × valence × ERQ suppression	4.82	4	256	0.45	0.772	0.007
	Block × ERQ reappraisal × ERQ suppression	11.21	2	256	2.10	0.125	0.016
	Valence × ERQ reappraisal × ERQ suppression	4.27	2	256	0.80	0.450	0.006
	Block × valence × ERQ reappraisal × ERQ suppression	3.84	4	256	0.36	0.838	0.006
	*R*^2^ marginal/conditional	0.09/0.31					

For each dependent variable (DV) and effect of interest, we report the sum of squares (SS), numerator (df_num_), and denominator (df_den_) degrees of freedom, F-test and relative p-values, and partial η^2^.

In the early time window (1,500–2,000 ms to S1 onset), significant relationships between ERQ scores and the early l-SMA and l-dPCC activity were observed. In particular, as for the early l-SMA activity, we found an interaction between block and the ERQ suppression subscale [*F*_(2, 256)_ = 3.26, *p* = 0.04; see Figure 2A]. The slope analysis suggested that higher suppression scores were associated with a reduced l-SMA activity in the 100 and 50% blocks (contrary to H2), and with a higher l-SMA activity in the 75% block (consistently with H2). However, no slope was statistically different from 0 (see Supplementary Table 3). *Post-hoc* contrasts showed evidence of a difference in the slopes between the 75 and 50% blocks [75 vs. 50%: *t*_(256)_ = 2.39, SE = 0.16, *p* = 0.046]. Moreover, as for the early l-dPCC activity, we found an interaction among block, the ERQ reappraisal, and the ERQ suppression subscales [*F*_(2, 256)_ = 3.65, *p* = 0.027]. However, neither slope analysis nor *post-hoc* contrasts showed significant effects (see Supplementary Table 3).

In the late time window (2,000–2,500 ms to S1 onset), significant relationships between ERQ scores and the late CNV amplitude were found. In particular, a significant interaction between block and the suppression subscale emerged [*F*_(2, 256)_ = 3.67, *p* = 0.027; see Figure 2B]. The slope analysis suggested that higher suppression scores were associated with a smaller late CNV amplitude in the 75 and 50% blocks (contrary to H2) vs. a larger late CNV amplitude in the 100% block (consistently with H2). However, no slope was statistically different from 0 (see Supplementary Table 3). *Post-hoc* contrasts showed evidence of a difference in the slopes between the 100 and 75% blocks [100 vs. 75%: *t*_(256)_ = −2.71, SE = 0.12, *p* = 0.02].

### Prediction updating stage

**Figure 3:**
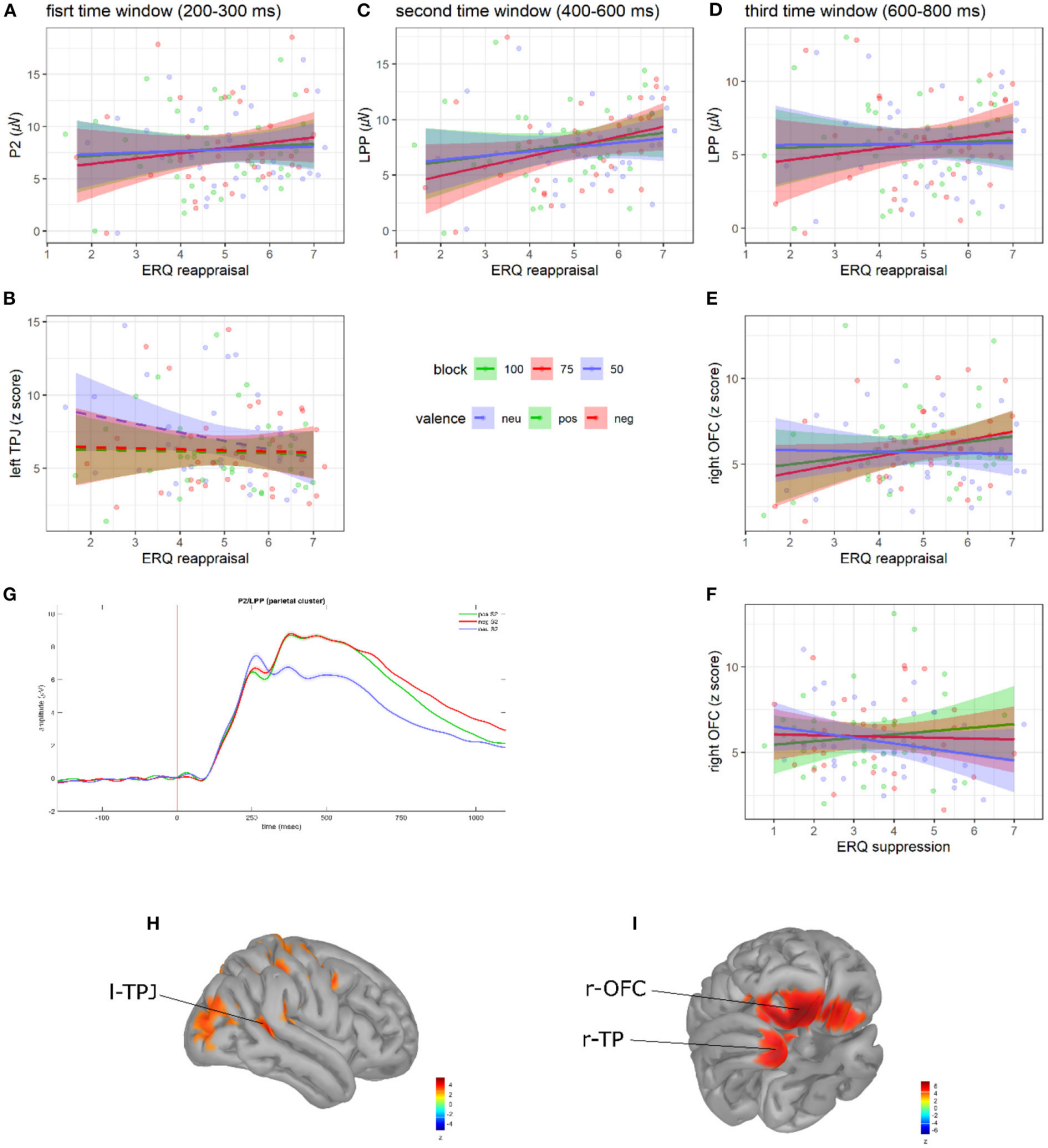
Regression plots of ERQ reappraisal scores on **(A)** P2 amplitude, **(C)** early LPP amplitude, **(D)** late LPP amplitude, and **(E)** late r-OFC activity as a function of blocks. Regression plot of ERQ reappraisal scores on **(B)** l-TPJ activity as a function of S2 valence. Regression plot of ERQ suppression scores on **(F)** late r-OFC activity as a function of blocks. Shaded areas denote the 95% CI. Points represent individual observations. Grand average ERP waveforms to S2s **(G)**. Shaded areas denote SE. Adapted from Del Popolo Cristaldi et al. (2021d). Brain activity (*z*-score) of the l-TPJ **(H)**, r-OFC, and r-TP **(I)**.

Models on P2, l-TPJ, r-TPJ, LPP, r-OFC, and r-TP activity are summarized in Table 3, Figure 3, and Supplementary Table 4.

**Table 3:** ANOVA models in the prediction updating stage.

**DV**	**Effect**	**SS**	**df_num_**	**df_den_**	***F***	***p***	**Partial η^2^**
P2	Block	1.32	2	256	0.41	0.664	0.003
	Valence	74.68	2	256	23.22	<0.001	0.154
	ERQ reappraisal	0.50	1	32	0.31	0.581	0.010
	ERQ suppression	0.28	1	32	0.17	0.681	0.005
	Block × valence	3.47	4	256	0.54	0.707	0.008
	Block × ERQ reappraisal	14.68	2	256	4.56	0.011	0.034
	Valence × ERQ reappraisal	2.20	2	256	0.69	0.505	0.005
	Block × ERQ suppression	2.53	2	256	0.79	0.457	0.006
	Valence × ERQ suppression	5.12	2	256	1.59	0.205	0.012
	ERQ reappraisal × ERQ suppression	6.45	1	32	4.01	0.054	0.111
	Block × valence × ERQ reappraisal	3.67	4	256	0.57	0.685	0.009
	Block × valence × ERQ suppression	7.17	4	256	1.11	0.350	0.017
	Block × ERQ reappraisal × ERQ suppression	0.02	2	256	0.01	0.993	0.000
	Valence × ERQ reappraisal × ERQ suppression	4.05	2	256	1.26	0.286	0.010
	Block × valence × ERQ reappraisal × ERQ suppression	3.58	4	256	0.56	0.694	0.009
	*R*^2^ marginal/conditional	0.12/0.91					
r-TPJ	Block	11.50	2	256	1.30	0.275	0.010
	Valence	67.88	2	256	7.66	<0.001	0.056
	ERQ reappraisal	0.28	1	32	0.06	0.804	0.002
	ERQ suppression	3.72	1	32	0.84	0.366	0.026
	Block × valence	23.52	4	256	1.33	0.260	0.020
	Block × ERQ reappraisal	0.38	2	256	0.04	0.958	0.000
	Valence × ERQ reappraisal	7.61	2	256	0.86	0.425	0.007
	Block × ERQ suppression	3.60	2	256	0.41	0.667	0.003
	Valence × ERQ suppression	3.50	2	256	0.40	0.674	0.003
	ERQ reappraisal × ERQ suppression	3.36	1	32	0.76	0.390	0.023
	Block × valence × ERQ reappraisal	8.19	4	256	0.46	0.763	0.007
	Block × valence × ERQ suppression	8.21	4	256	0.46	0.763	0.007
	Block × ERQ reappraisal × ERQ suppression	0.94	2	256	0.11	0.899	0.001
	Valence × ERQ reappraisal × ERQ suppression	4.99	2	256	0.56	0.570	0.004
	Block × valence × ERQ reappraisal × ERQ suppression	6.36	4	256	0.36	0.838	0.006
	*R*^2^ marginal/conditional	0.07/0.64					
l-TPJ	Block	35.21	2	256	4.01	0.019	0.030
	Valence	39.48	2	256	4.49	0.012	0.034
	ERQ reappraisal	1.93	1	32	0.44	0.512	0.014
	ERQ suppression	3.74	1	32	0.85	0.363	0.026
	Block × valence	23.59	4	256	1.34	0.255	0.021
	Block × ERQ reappraisal	3.32	2	256	0.38	0.686	0.003
	Valence × ERQ reappraisal	36.95	2	256	4.21	0.016	0.032
	Block × ERQ suppression	16.49	2	256	1.88	0.155	0.014
	Valence × ERQ suppression	4.93	2	256	0.56	0.571	0.004
	ERQ reappraisal × ERQ suppression	0.53	1	32	0.12	0.731	0.004
	Block × valence × ERQ reappraisal	4.68	4	256	0.27	0.899	0.004
	Block × valence × ERQ suppression	10.27	4	256	0.58	0.674	0.009
	Block × ERQ reappraisal × ERQ suppression	2.18	2	256	0.25	0.78	0.002
	Valence × ERQ reappraisal × ERQ suppression	2.94	2	256	0.33	0.716	0.003
	Block × valence × ERQ reappraisal × ERQ suppression	6.89	4	256	0.39	0.814	0.006
	*R*^2^ marginal/conditional	0.07/0.66					
Early LPP	Block	3.33	2	256	0.94	0.391	0.007
	Valence	361.91	2	256	102.37	<0.001	0.444
	ERQ reappraisal	3.32	1	32	1.88	0.180	0.055
	ERQ suppression	0.34	1	32	0.19	0.664	0.006
	Block × valence	1.92	4	256	0.27	0.897	0.004
	Block × ERQ reappraisal	28.40	2	256	8.03	<0.001	0.059
	Valence × ERQ reappraisal	8.77	2	256	2.48	0.086	0.019
	Block × ERQ suppression	2.38	2	256	0.67	0.511	0.005
	Valence × ERQ suppression	6.07	2	256	1.72	0.182	0.013
	ERQ reappraisal × ERQ suppression	8.21	1	32	4.65	0.039	0.127
	Block × valence × ERQ reappraisal	6.13	4	256	0.87	0.484	0.013
	Block × valence × ERQ suppression	4.09	4	256	0.58	0.679	0.009
	Block × ERQ reappraisal × ERQ suppression	8.93	2	256	2.53	0.082	0.019
	Valence × ERQ reappraisal × ERQ suppression	5.17	2	256	1.46	0.234	0.011
	Block × valence × ERQ reappraisal × ERQ suppression	6.10	4	256	0.86	0.487	0.013
	*R*^2^ marginal/conditional	0.22/0.89					
Early r-OFC	Block	8.37	2	256	0.90	0.409	0.007
	Valence	486.06	2	256	52.11	<0.001	0.289
	ERQ reappraisal	13.87	1	32	2.98	0.094	0.085
	ERQ suppression	0.04	1	32	0.01	0.927	0.000
	Block × valence	14.80	4	256	0.79	0.530	0.012
	Block × ERQ reappraisal	25.38	2	256	2.72	0.068	0.021
	Valence × ERQ reappraisal	19.86	2	256	2.13	0.121	0.016
	Block × ERQ suppression	12.39	2	256	1.33	0.267	0.010
	Valence × ERQ suppression	4.86	2	256	0.52	0.595	0.004
	ERQ reappraisal × ERQ suppression	0.06	1	32	0.01	0.909	0.000
	Block × valence × ERQ reappraisal	17.84	4	256	0.96	0.432	0.015
	Block × valence × ERQ suppression	19.55	4	256	1.05	0.383	0.016
	Block × ERQ reappraisal × ERQ suppression	2.45	2	256	0.26	0.769	0.002
	Valence × ERQ reappraisal × ERQ suppression	0.86	2	256	0.09	0.912	0.001
	Block × valence × ERQ reappraisal × ERQ suppression	12.77	4	256	0.68	0.603	0.011
	*R*^2^ marginal/conditional	0.20/0.62					
Early r-TP	Block	24.10	2	256	1.86	0.158	0.014
	Valence	347.75	2	256	26.78	<0.001	0.173
	ERQ reappraisal	1.88	1	32	0.29	0.594	0.009
	ERQ suppression	0.44	1	32	0.07	0.797	0.002
	Block × Valence	17.31	4	256	0.67	0.616	0.010
	Block × ERQ reappraisal	9.83	2	256	0.76	0.470	0.006
	Valence × ERQ reappraisal	5.81	2	256	0.45	0.640	0.003
	Block × ERQ suppression	14.07	2	256	1.08	0.340	0.008
	Valence × ERQ suppression	23.71	2	256	1.83	0.163	0.014
	ERQ reappraisal × ERQ suppression	5.13	1	32	0.79	0.381	0.024
	Block × Valence × ERQ reappraisal	19.38	4	256	0.75	0.561	0.012
	Block × Valence × ERQ suppression	16.68	4	256	0.64	0.633	0.010
	Block × ERQ reappraisal × ERQ suppression	2.97	2	256	0.23	0.796	0.002
	Valence × ERQ reappraisal × ERQ suppression	0.51	2	256	0.04	0.962	0.000
	Block × Valence × ERQ reappraisal × ERQ suppression	21.63	4	256	0.83	0.505	0.013
	R^2^ marginal/conditional	0.11/0.60					
Late LPP	Block	0.22	2	256	0.06	0.941	0.000
	Valence	349.53	2	256	95.29	<0.001	0.427
	ERQ reappraisal	0.24	1	32	0.13	0.720	0.004
	ERQ suppression	1.12	1	32	0.61	0.440	0.019
	Block × Valence	6.79	4	256	0.93	0.449	0.014
	Block × ERQ reappraisal	15.66	2	256	4.27	0.015	0.032
	Valence × ERQ reappraisal	5.75	2	256	1.57	0.210	0.012
	Block × ERQ suppression	1.32	2	256	0.36	0.699	0.003
	Valence × ERQ suppression	7.01	2	256	1.91	0.150	0.015
	ERQ reappraisal × ERQ suppression	9.05	1	32	4.93	0.034	0.134
	Block × valence × ERQ reappraisal	8.98	4	256	1.22	0.301	0.019
	Block × valence × ERQ suppression	3.77	4	256	0.51	0.726	0.008
	Block × ERQ reappraisal × ERQ suppression	10.57	2	256	2.88	0.058	0.022
	Valence × ERQ reappraisal × ERQ suppression	4.41	2	256	1.20	0.303	0.009
	Block × valence × ERQ reappraisal × ERQ suppression	6.75	4	256	0.92	0.453	0.014
	*R*^2^ marginal/conditional	0.21/0.85					
Late r-OFC	Block	4.96	2	256	0.50	0.608	0.004
	Valence	468.57	2	256	47.09	<0.001	0.269
	ERQ reappraisal	6.08	1	32	1.22	0.277	0.037
	ERQ suppression	0.31	1	32	0.06	0.805	0.002
	Block × valence	11.06	4	256	0.56	0.695	0.009
	Block × ERQ reappraisal	30.29	2	256	3.04	0.049	0.023
	Valence × ERQ reappraisal	3.14	2	256	0.32	0.729	0.002
	Block × ERQ suppression	30.56	2	256	3.07	0.048	0.023
	Valence × ERQ suppression	2.33	2	256	0.23	0.791	0.002
	ERQ reappraisal × ERQ suppression	0.03	1	32	0.01	0.943	0.000
	Block × valence × ERQ reappraisal	11.32	4	256	0.57	0.686	0.009
	Block × valence × ERQ suppression	17.90	4	256	0.90	0.465	0.014
	Block × ERQ reappraisal × ERQ suppression	2.24	2	256	0.23	0.799	0.002
	Valence × ERQ reappraisal × ERQ suppression	5.93	2	256	0.60	0.552	0.005
	Block × valence × ERQ reappraisal × ERQ suppression	22.75	4	256	1.14	0.337	0.018
	*R*^2^ marginal/conditional	0.20/0.50					
Late r-TP	Block	13.50	2	256	1.15	0.317	0.009
	Valence	387.36	2	256	33.11	<0.001	0.206
	ERQ reappraisal	0.51	1	32	0.09	0.769	0.003
	ERQ suppression	1.36	1	32	0.23	0.633	0.007
	Block × valence	11.85	4	256	0.51	0.731	0.008
	Block × ERQ reappraisal	9.24	2	256	0.79	0.455	0.006
	Valence × ERQ reappraisal	0.40	2	256	0.03	0.966	0.000
	Block × ERQ suppression	6.22	2	256	0.53	0.588	0.004
	Valence × ERQ suppression	24.34	2	256	2.08	0.127	0.016
	ERQ reappraisal × ERQ suppression	4.08	1	32	0.70	0.410	0.021
	Block × valence × ERQ reappraisal	19.54	4	256	0.83	0.504	0.013
	Block × valence × ERQ suppression	11.55	4	256	0.49	0.740	0.008
	Block × ERQ reappraisal × ERQ suppression	1.86	2	256	0.16	0.853	0.001
	Valence × ERQ reappraisal × ERQ suppression	1.83	2	256	0.16	0.856	0.001
	Block × valence × ERQ reappraisal × ERQ suppression	17.08	4	256	0.73	0.572	0.011
	*R*^2^ marginal/conditional	0.15/0.50					

For each dependent variable (DV) and effect of interest, we report the sum of squares (SS), numerator (df_num_), and denominator (df_den_) degrees of freedom, F-test and relative p-values, and partial η^2^.

In the first time window (200–300 ms to S2 onset), significant relationships between ERQ scores and both P2 amplitude and l-TPJ activity were observed. As for P2 amplitude, we found an interaction between block and the ERQ reappraisal subscale [*F*_(2, 256)_ = 4.56, *p* = 0.011; see Figure 3A]. As hypothesized (H3), the slope analysis suggested that higher reappraisal scores were associated with increased P2 amplitude in all the blocks (but with a steeper slope in the 75% block). However, no slope was statistically different from 0 (see Supplementary Table 4). *Post-hoc* contrasts showed evidence of a difference in the slopes between the 75 and 50% blocks [75 vs. 50%: *t*_(256)_ = 2.88, SE = 0.12, *p* = 0.012]. As for l-TPJ activity, instead, we found an interaction between valence and the ERQ reappraisal subscale [*F*_(2, 256)_ = 4.21, *p* = 0.016; see Figure 3B]. The slope analysis suggested that higher reappraisal scores were associated with a reduced l-TPJ activity to S2s, especially those with neutral valence. However, no slope was statistically different from 0 (see Supplementary Table 4). *Post-hoc* contrasts showed evidence of a difference in the slopes between neutral and both negative and positive S2s [NEU vs. POS: *t*_(256)_ = −2.52, SE = 0.21, *p* = 0.033; NEU vs. NEG: *t*_(256)_ = −2.5, SE = 0.21, *p* = 0.035].

In the second time window (400–600 ms to S2 onset), significant relationships between ERQ scores and early LPP amplitude emerged. In particular, we found an interaction between block and the ERQ reappraisal subscale [*F*_(2, 256)_ = 8.03, *p* <0.001; see Figure 3C]. As hypothesized (H3), the slope analysis revealed that higher reappraisal scores predicted larger LPP amplitudes in all the blocks, but with a slope statistically different from 0 only in the 75% block [*b* = 0.85, *b*_SE_ = 0.41, 95% CI = (0.01, 1.69)]. From *post-hoc* contrasts, it emerged that the slope in the 75% block was significantly different than in both 100 and 50% blocks [100 vs. 75%: *t*_(256)_ = −2.86, SE = 0.13, *p* = 0.013; 75 vs. 50%: *t*_(256)_ = 3.86, SE = 0.13, *p* <0.001].

In the third time window (600–800 ms to S2 onset), significant relationships between ERQ scores and both late LPP amplitude and r-OFC activity were observed. As for the late LPP amplitude, a significant interaction between block and the ERQ reappraisal subscale [*F*_(2, 256)_ = 4.27, *p* = 0.015; see Figure 3D] was found. The slope analysis showed that higher reappraisal scores predicted a larger late LPP amplitude in the 100 and 75% block (consistently with H3), and slightly smaller LPP in the 50% block (contrary to H3). However, no slope was statistically different from 0 (see Supplementary Table 4). *Post-hoc* comparisons showed a significant difference in the slopes between the 75 and 50% blocks [75 vs. 50%: *t*_(256)_ = 2.76, SE = 0.14, *p* = 0.017]. As for the r-OFC, instead, significant interactions were found between block and both the ERQ reappraisal [*F*_(2, 256)_ = 3.04, *p* = 0.049; see Figure 3E] and suppression subscales [*F*_(2, 256)_ = 3.07, *p* = 0.048; see Figure 3F]. The slope analysis revealed positive relationships between reappraisal scores and r-OFC activity in the 100 and 75% blocks (consistently with H3) and negative relationships in the 50% block (contrary to H3), whereas suppression scores showed a positive relationship in the 100% block and negative relationships in the 75 and 50% blocks. However, no slope was statistically different from 0 (see Supplementary Table 4). *Post-hoc* contrasts showed significant block differences in the relationship between r-OFC activity and both reappraisal scores in the 75 and 50% blocks [75 vs. 50%: *t*_(256)_ = 2.4, SE = 0.22, *p* = 0.045], and suppression scores in the 100 and 50% blocks [100 vs. 50%: *t*_(256)_ = 2.47, SE = 0.22, *p* = 0.037].

## Discussion

To our knowledge, this study is the first to provide evidence that habitual ER strategies interact with contextual uncertainty in modulating the neural correlates of affective predictions at both the scalp and source levels. As the two main findings, our results suggest that expressive suppression and cognitive reappraisal are differently deployed depending on the specific prediction stage (generation-implementation-updating) and that both strategies interact with the levels of contextual uncertainty by differently modulating ERPs and source activity within each stage.

Concerning the first finding, we found that neural activity in the generation stage was not modulated by any ER strategy. However, suppression and reappraisal uniquely mediated ERPs and source activations in the implementation and updating stages, respectively. This is consistent with our hypotheses (H1, H2, and H3) and the majority of extant studies (Ochsner et al., 2002; Herwig et al., 2007; Bernat et al., 2011; Herbert et al., 2013; Shimamura et al., 2013; Vanderhasselt et al., 2013a,b; Wu et al., 2013; Baur et al., 2015; Gan et al., 2015; Paul et al., 2016; Langeslag and Surti, 2017; Shafir and Sheppes, 2018; Myruski et al., 2019; Zhu et al., 2019; Cao et al., 2020; MacNamara et al., 2022), as well as with what can be expected integrating the predictions of the process model of ER (Gross, 1998; Gross and John, 2003) and predictive models of emotion (Seth and Friston, 2016; Barrett, 2017). During the generation stage, indeed, it is crucial to achieve an efficient evaluation of the available environmental cues. Those cues are then used in combination with previous experience to constrain and refine the pool of information used to generate affective predictive models (Knill and Pouget, 2004; Bar, 2007; Seth and Friston, 2016; Shipp, 2016; Barrett, 2017). Thus, when presented with a cue conveying information about forthcoming (and potentially relevant) stimuli, it may be more efficient to extract as much information as possible (and as early as possible) from the cue itself, before trying to regulate its affective impact. Accordingly, in our paradigm, no ER strategy appeared to mediate the quick extraction of affective and predictive information from the faces (S1s), as reflected by the N170 amplitude and r-STS activity.

During the implementation stage, instead, it becomes primary to program and prepare the best action plans and to anticipate the physiological changes to deal with the predicted stimulus/situation (van Boxtel and Böcker, 2004; Seth and Friston, 2016; Barrett, 2017). Here, we found the only involvement of the suppression ER strategy, which predicted an early (1,500–2,000 ms to S1 onset) increase of the left SMA activity, and a later (2,000–2,500 ms) decrease of the CNV amplitude in the moderately predictive context (75% block). These effects may reflect the intention to suppress the affective impact of the forthcoming S2. The increased SMA activity is consistent with evidence showing its left-lateralized involvement in ER (Morawetz et al., 2017). It could be related to the proactive deployment of anticipatory inhibitory control processes (Vanderhasselt et al., 2013b), which are proven to play a critical role in the expressive suppression of emotions (Garavan et al., 2006; Lee et al., 2008; Kunz et al., 2011). The reduced allocation of anticipatory resources reflected by a smaller CNV could complement the processes underpinned by the SMA activation, reducing the sustained scanning of concurrent environmental information and eventually serving an attempt to avoid distractions or interference on the suppression process.

During prediction updating, finally, actual inputs (i.e., S2s) must be compared with predictions in order to adjust the predictive models according to the actual evidence (Friston, 2010; Seth and Friston, 2016; Barrett, 2017). Thus, it seems crucial to reappraise the S2 to diminish its affective impact, and to maximize the processing and encoding of the information, it carries about the match/mismatch with ongoing predictions. By dampening the affective impact of the S2, an efficient updating of the affective predictive model may be ultimately facilitated. Coherently, in our paradigm, we found a major involvement of the reappraisal ER strategy in the modulation of S2-locked neural activity. In particular, results showed that cognitive reappraisal predicted larger P2 and early LPP amplitudes in all the predictive contexts, and increased late LPP and r-OFC activity in the fully (100%) and moderately predictive (75%) contexts. These results are consistent with the literature suggesting that reappraisal elicits larger LPP amplitudes when the stimuli to be reappraised are highly arousing, like those we employed in our paradigm (Hajcak et al., 2010; Langeslag and Surti, 2017). Within a predictive framework, these results could index the involvement of cognitive reappraisal in supporting the updating of predictive models (as reflected by the P2) in light of the information derived from a deep S2 processing (as reflected by the LPP and r-OFC activity). Moreover, the involvement of the right OFC is consistent with extant evidence suggesting that reappraisal is uniquely associated with OFC activation (Dörfel et al., 2014), especially when a down-regulation is achieved (Ochsner et al., 2004; Kim and Hamann, 2007). Interestingly, in a later time window (600–800 ms from S2), these processes seem to persist only in those predictive contexts in which the S2 frequently matches predictions (100 and 75%). It is indeed cognitively and metabolically inefficient to carry on a sustained S2 processing when it disconfirms predictions so often (as in the 50% block), thus becoming totally uninformative with respect to adjusting future predictions.

As a second main finding, we found that both suppression and reappraisal strategies interacted with the levels of contextual uncertainty by differently modulating ERPs and source activity within each stage. These results offer at least two important contributions. First, the majority of the significant effects involved an interaction between ERQ scores and blocks. In particular, regression plots and *post-hoc* contrasts clearly showed that in the implementation stage and in the latest time window of the updating stage the relationships between ER strategies and neural activity presented distinct patterns depending on the predictive context. This highlights the importance of taking into account the role of contextual uncertainty when investigating the relationships between ER and affective processing. Second, from the overall pattern of results, the 75% block emerged as the one showing the most significant comparisons. This suggests that ER strategies predominantly modulate neural activity in those conditions where they most support an efficient updating of affective predictive models. The 75% block is the only one in which model updating occurs, since it implies expectancy violation in incongruent trials. In the 100 and 50% blocks, instead, no model updating occurs since they both involve no expectancy violation (in the 100% block because there are no incongruent trials, while in the 50% block because it is impossible to generate any reliable predictive model). Thus, the moderately predictive condition seems best suited to study affective prediction construction and its interactions with ER strategies. Moreover, it also closely resembles the contingencies we are most exposed to in everyday life.

It is worth commenting briefly on two unexpected results regarding the updating stage. First, we found that cognitive reappraisal negatively predicted the left TPJ activity to neutral S2s. This is surprising, considering that in our previous works (Del Popolo Cristaldi et al., 2021c,d), the modulation of the TPJ was consistent with the direction of the modulation of the P2 (i.e., larger P2 amplitudes corresponded to higher TPJ activity), and that in the present work, no interactions between reappraisal scores and S2 valence emerged on the P2. Since the TPJ is involved in contextual updating (Geng and Vossel, 2013) and the left TPJ is generally involved in ER (Morawetz et al., 2017), we might speculate that a reduced l-TPJ activation to neutral S2s may reflect the fact that neutral stimuli require a reduced reappraisal effort (they are less salient than emotional stimuli). Second, in the late time window (600–800 ms from S2), r-OFC activity was found to be significantly predicted not only by reappraisal, but also by suppression scores. Interestingly, the direction of the relationship between expressive suppression and r-OFC activity was the same as for cognitive reappraisal in 100 and 50% blocks. Both suppression and reappraisal scores predicted higher r-OFC activity in the 100% block and lower r-OFC activity in the 50% block. However, an opposite pattern emerged in the 75% block, with reappraisal predicting higher and suppression predicting lower r-OFC activity. Thus, it seems that in a later time window the suppression strategy is mobilized in addition to reappraisal, summing its effect in those contexts in which no model updating is required (100 and 50% block) and promoting an opposite effect in the context requiring model updating (75% block). However, it is important to note that these unexpected results may be due to spurious effects. Thus, any interpretation remains tentative and speculative, and future studies are needed to better clarify these effects.

As important limitations to be acknowledged, we have to mention first a limited sample size, that may have negatively impacted the generalizability of results. Moreover, slope analysis revealed that the majority of the slopes were not significantly different from 0. Therefore, these results do not appear to be highly robust, and their interpretation should be considered with caution. Lastly, in this study, we investigated the effect of habitual ER strategies, while no measure or manipulation of the context-based, circumscribed ER strategies has been carried out. However, these two facets of emotion regulation (i.e., habitual vs. context-based use of ER strategies) may interact or overlap, and further studies are needed in order to disentangle any potential mutual influence.

Despite these limitations, our study nonetheless provides interesting insights for present and future research. To summarize, we provided evidence that expressive suppression and cognitive reappraisal are differently deployed depending on the specific stage in which affective prediction unfolds. Furthermore, we demonstrated that both strategies interact with contextual uncertainty by differentially modulating the neural correlates of affective predictions within the implementation and updating stages. As a first contribution, our results offer a nice framework to study ER strategies and their influence on affective processing, by combining insights from the process model of ER (Gross, 1998; Gross and John, 2003) with others from predictive models of emotion (Seth and Friston, 2016; Barrett, 2017). Second, given the role of altered ER (Cisler et al., 2010) and interoceptive prediction patterns (Brewer et al., 2021) as risk factors for the development of affective psychopathology, our results may provide some potential clinical implications. The assessment of the influence of ER strategies on affective processing through paradigms similar to ours could, for instance, be included among clinical practices to early detect people at risk of psychopathology. Or, clinical interventions that train the circumstantial use of the most efficient ER strategies could be implemented, and compensate for the habitual use of ineffective strategies that are typical of individuals with manifest psychopathology.

## Data availability statement

Publicly available datasets from the Figshare repository were analyzed in this study. This data can be found here: https://doi.org//10.6084/m9.figshare.13560569.

## Ethics statement

The study was reviewed and approved by Ethical Committee for the Psychological Research of the University of Padua (protocol no. 2859), and it was conducted in accordance with the Declaration of Helsinki. All the participants provided their written informed consent to participate in this study.

## Author contributions

FDPC: conceptualization, methodology, formal analysis, investigation, data curation, writing—original draft, writing—review and editing, and visualization. GM and GB: conceptualization and methodology. MS: conceptualization, methodology, writing—review and editing, supervision, and project administration. All authors contributed to the article and approved the submitted version.

## Funding

The study was supported by a grant from MIUR (Dipartimenti di Eccellenza DM 11/05/2017 n. 262) to the Department of General Psychology.

## Conflict of interest

The authors declare that the research was conducted in the absence of any commercial or financial relationships that could be construed as a potential conflict of interest.

## Publisher's note

All claims expressed in this article are solely those of the authors and do not necessarily represent those of their affiliated organizations, or those of the publisher, the editors and the reviewers. Any product that may be evaluated in this article, or claim that may be made by its manufacturer, is not guaranteed or endorsed by the publisher.
