# Supplementary material for: Emotion regulation strategies differentially modulate neural activity across affective prediction stages: An HD-EEG investigation
Source: Front Behav Neurosci. 2022 Aug 5;16:947063. doi: 10.3389/fnbeh.2022.947063 (PMC9388773; doi:10.3389/fnbeh.2022.947063)
Supplement: Supplementary file 1 [file Data_Sheet_1.PDF]

SUPPLEMENTARY MATERIAL

Emotion regulation strategies differentially modulate neural activity across affective prediction stages: an HD-EEG investigation

Fiorella Del Popolo Cristaldi[1], Giovanni Mento[1,2], Giulia Buodo[1], Michela Sarlo[3]

[1]Department of General Psychology, University of Padua, Padua, Italy
[2]Padua Neuroscience Center (PNC), University of Padua, Padua, Italy
[3]Department of Communication Sciences, Humanities and International Studies, University of Urbino Carlo Bo, Urbino, Italy

Table S1 List of NimStim and IAPS picture numbers used as S1s and S2s, sorted by valence. POS = positive, NEG = negative, NEU = neutral

Valence	NimStim	IAPS
POS	01F_HA_O; 03F_HA_O; 06F_HA_O; 09F_HA_O; 28M_HA_O; 33M_HA_O; 34M_HA_O; 36M_HA_O	4647; 4651; 4652; 4653; 4656; 4658; 4659; 4664; 4666; 4669; 4670; 4672; 4680; 4683; 4687; 4690; 4694; 4695; 4800; 4810; 5621; 8021; 8030; 8031; 8034; 8040; 8080; 8160; 8161; 8178; 8179; 8180; 8185; 8186; 8193; 8200; 8210; 8370; 8400; 8490
NEG	01F_FE_O; 03F_FE_O; 06F_FE_O; 09F_FE_O; 28M_FE_O; 33M_FE_O; 34M_FE_O; 36M_FE_O	3000; 3010; 3015; 3030; 3051; 3053; 3060; 3068; 3071; 3080; 3100; 3102; 3110; 3120; 3130; 3140; 3150; 3400; 3550; 6190; 6200; 6210; 6211; 6213; 6230; 6242; 6243; 6250; 6260; 6300; 6312; 6313; 6315; 6350; 6360; 6510; 6530; 6540; 6550; 9405
NEU	01F_NE_O; 03F_NE_O; 06F_NE_O; 09F_NE_O; 28M_NE_O; 33M_NE_O; 34M_NE_O; 36M_NE_O	7000; 7002; 7004; 7006; 7009; 7010; 7020; 7025; 7030; 7031; 7034; 7035; 7036; 7037; 7039; 7040; 7041; 7050; 7052; 7056; 7060; 7080; 7090; 7100; 7110; 7130; 7140; 7150; 7170; 7175; 7211; 7217; 7224; 7233; 7234; 7235; 7491; 7495; 7500; 7510

Table S2 Means (*M*), standard deviations (*SD*), test statistics (*F*), and associated *p*-values (*p*) of the final number of epochs accepted for each experimental condition.
Results showed no significant differences between conditions.

**Block**	**100%**		**75%**		**50%**		***F*(2,105)**	***p***
	M	*SD*	*M*	*SD*	*M*	*SD*	0.42	0.66
	113.33	5.87	114.56	6.39	114.17	5.07		
**Valence**	**POS**		**NEG**		**NEU**		***F*(2, 105)**	***p***
S1	*M*	*SD*	*M*	*SD*	*M*	*SD*	0.52	0.6
	113.64	4.87	114.67	4.34	113.75	4.90		
S2	*M*	*SD*	*M*	*SD*	*M*	*SD*	0.26	0.78
	113.72	4.93	114.47	4.62	113.86	4.64		

Table S3 Slope analysis and post-hoc contrasts of the significant effects of models in the prediction implementation stage. For each dependent variable (DV) the significant effect is reported in parentheses.
SE = standard error, 95% CI = 95% confidence interval, df = degrees of freedom

**DV (effect)**	**Slope analysis**			
early l-SMA (block x ERQ suppression)	**Block**	***b***	b_{SE}	**95% CI**
	100%	-0.11	0.14	-0.39, 0.18
	75%	0.21	0.14	-0.08, 0.49
	50%	-0.17	0.14	-0.46, 0.11
	**Post-hoc contrasts**			
	**Contrast**	***t*(df)**	**SE**	***p***
	100% vs. 75%	-1.97(256)	0.16	0.122
	100% vs. 50%	0.42(256)	0.16	0.907

	75% vs. 50%	2.39(256)	0.16	0.046
early l-dPCC (block x ERQ reappraisal)	**Slope analysis**			
	**Block**	***b***	**b_{SE}**	**95% CI**
	100%	0.20	0.14	-0.08, 0.48
	75%	-0.03	0.14	-0.31, 0.25
	50%	0.20	0.14	-0.08, 0.48
	**Post-hoc contrasts**			
	**Contrast**	***t*(df)**	**SE**	***p***
	100% vs. 75%	1.61(256)	0.14	0.243
	100% vs. 50%	0.02(256)	0.14	0.999
	75% vs. 50%	-1.59(256)	0.14	0.252
early l-dPCC (block x ERQ suppression)	**Slope analysis**			
	**Block**	***b***	**b_{SE}**	**95% CI**
	100%	0.14	0.14	-0.14, 0.41
	75%	0.15	0.14	-0.13, 0.42
	50%	-0.07	0.14	-0.35, 0.20
	**Post-hoc contrasts**			
	**Contrast**	***t*(df)**	**SE**	***p***
	100% vs. 75%	0.07(256)	0.14	0.997
	100% vs. 50%	1.52(256)	0.14	0.283
	75% vs. 50%	1.59(256)	0.14	0.252
late CNV (block x ERQ suppression)	**Slope analysis**			
	**Block**	***b***	**b_{SE}**	**95% CI**
	100%	-0.13	0.1	-0.33, 0.07
	75%	0.19	0.1	-0.01, 0.39
	50%	0.04	0.1	-0.16, 0.24
	**Post-hoc contrasts**			
	**Contrast**	***t*(df)**	**SE**	***p***
	100% vs. 75%	-2.71(256)	0.12	0.02
	100% vs. 50%	-1.41(256)	0.12	0.338
	75% vs. 50%	1.3(256)	0.12	0.397

Table S4 Slope analysis and post-hoc contrasts of the significant effects of models in the prediction updating stage. For each dependent variable (DV) the significant effect is reported in parentheses.
SE = standard error, 95% CI = 95% confidence interval, df = degrees of freedom

**DV (effect)**	**Slope analysis**			
P2 (block x ERQ reappraisal)	**Block**	***b***	**b_{SE}**	**95% CI**
	100%	0.19	0.46	-0.74, 1.11
	75%	0.47	0.46	-0.46, 1.39
	50%	0.1	0.46	-0.83, 1.03
	**Post-hoc contrasts**			
	**Contrast**	***t*(df)**	**SE**	***p***
	100% vs. 75%	-2.22(256)	0.13	0.07
	100% vs. 50%	0.66(256)	0.13	0.785
	75% vs. 50%	2.88(256)	0.13	0.012
l-TPJ (valence x ERQ reappraisal)	**Slope analysis**			
	**Valence**	***b***	**b_{SE}**	**95% CI**
	NEU	-0.58	0.37	-1.32, 0.16
	POS	-0.05	0.37	-0.79, 0.69
	NEG	-0.06	0.37	-0.79, 0.68
	**Post-hoc contrasts**			
	**Contrast**	***t*(df)**	**SE**	***p***
	NEU vs. POS	-2.52(256)	0.21	0.033
	NEU vs. NEG	-2.5(256)	0.21	0.035
	POS vs. NEG	0.02	0.21	0.999
early LPP (block x ERQ reappraisal)	**Slope analysis**			
	**Block**	***b***	**b_{SE}**	**95% CI**

	100%	0.47	0.41	-0.36, 1.31
	75%	0.85	0.41	0.01, 1.69
	50%	-0.5	0.41	-0.5, 1.18
	**Post-hoc contrasts**			
	**Contrast**	***t*(df)**	**SE**	***p***
	100% vs. 75%	-2.86(256)	0.13	0.013
	100% vs. 50%	1.00(256)	0.13	0.579
	75% vs. 50%	3.86	0.13	< 0.001
late LPP (block x ERQ reappraisal)	**Slope analysis**			
	**Block**	***b***	b_{SE}	**95% CI**
	100%	0.05	0.36	-0.68, 0.79
	75%	0.35	0.36	-0.38, 1.09
	50%	-0.02	0.36	-0.76, 0.71
	**Post-hoc contrasts**			
	**Contrast**	***t*(df)**	**SE**	***p***
	100% vs. 75%	-2.22	0.14	0.069
	100% vs. 50%	0.53	0.14	0.856
	75% vs. 50%	2.76	0.14	0.017
late r-OFC (block x ERQ reappraisal)	**Slope analysis**			
	**Block**	***b***	b_{SE}	**95% CI**
	100%	0.33	0.26	-0.2, 0.85
	75%	0.48	0.26	-0.04, 1.01
	50%	-0.05	0.26	-0.58, 0.48
	**Post-hoc contrasts**			
	**Contrast**	***t*(df)**	**SE**	***p***
	100% vs. 75%	-0.71	0.22	0.76
	100% vs. 50%	1.69	0.22	0.21
	75% vs. 50%	2.4	0.22	0.045
late r-OFC (block x ERQ suppression)	**Slope analysis**			
	**Block**	***b***	b_{SE}	**95% CI**
	100%	0.20	0.26	-0.31, 0.71
	75%	-0.04	0.26	-0.55, 0.47
	50%	-0.33	0.26	-0.84, 0.18
	**Post-hoc contrasts**			
	**Contrast**	***t*(df)**	**SE**	***p***
	100% vs. 75%	1.11	0.22	0.508
	100% vs. 50%	2.47	0.22	0.037
	75% vs. 50%	1.36	0.22	0.362
